# Hierarchical Synchronization Estimation of Low- and High-Order Functional Connectivity Based on Sub-Network Division for the Diagnosis of Autism Spectrum Disorder

**DOI:** 10.3389/fnins.2021.810431

**Published:** 2022-02-10

**Authors:** Feng Zhao, Zhongwei Han, Dapeng Cheng, Ning Mao, Xiaobo Chen, Yuan Li, Deming Fan, Peiqiang Liu

**Affiliations:** ^1^School of Computer Science and Technology, Shandong Technology and Business University, Yantai, China; ^2^Department of Radiology, Yantai Yuhuangding Hospital, Yantai, China; ^3^School of Management Science and Engineering, Shandong Technology and Business University, Yantai, China; ^4^School of Information Science and Technology, Qingdao University of Science and Technology, Qingdao, China

**Keywords:** functional connectivity network, resting-state functional magnetic resonance imaging, matrix variate normal distribution, autism spectrum disorder, hierarchical sub-network method

## Abstract

Functional connectivity network (FCN) calculated by resting-state functional magnetic resonance imaging (rs-fMRI) plays an increasingly important role in the exploration of neurologic and mental diseases. Among the presented researches, the method of constructing FCN based on Matrix Variate Normal Distribution (MVND) theory provides a novel perspective, which can capture both low- and high-order correlations simultaneously with a clear mathematical interpretability. However, when fitting MVND model, the dimension of the parameters (i.e., population mean and population covariance) to be estimated is too high, but the number of samples is relatively quite small, which is insufficient to achieve accurate fitting. To address the issue, we divide the brain network into several sub-networks, and then the MVND based FCN construction algorithm is implemented in each sub-network, thus the spatial dimension of MVND is reduced and more accurate estimates of low- and high-order FCNs is obtained. Furthermore, for making up the functional connectivity which is lost because of the sub-network division, the rs-fMRI mean series of all sub-networks are calculated, and the low- and high-order FCN across sub-networks are estimated with the MVND based FCN construction method. In order to prove the superiority and effectiveness of this method, we design and conduct classification experiments on ASD patients and normal controls. The experimental results show that the classification accuracy of “hierarchical sub-network method” is greatly improved, and the sub-network found most related to ASD in our experiment is consistent with other related medical researches.

## Introduction

Functional connectivity networks (FCN), usually calculated from resting-state functional magnetic resonance imaging (rs-fMRI), using blood oxygenation level dependent (BOLD) signals as neurophysiological indicators, are playing an increasingly important role in exploring the working mechanism of the brain and investigating the brain’s functional variations of some mental disorders, such as autism spectrum disorder (ASD) (Felouat and Oukid-Khouas, 2020; Sun et al., 2021), major depressive disorder (Mousavian et al., 2020), Alzheimer’s disease (Jones et al., 2012; Wang et al., 2017), and its early stage, i.e., mild cognitive impairment (Chen et al., 2016; Zhang et al., 2020), et al.

FCN is a weighted network based on the graph theory, which takes the regions of interest (ROIs) in the brain as the nodes, the correlation of the rs-fMRI time series between different ROIs as the functional connectivity (FC) and the FC strength as the weight of the edge (Smith et al., 2013). Among all the methods for FC estimation, the most classic and popular example is Pearson’s Correlation (PC) (Chen et al., 2016; Zhao et al., 2018; Sun et al., 2021). So far, it has been commonly known that the brain network structures and edge weights of the patients are different from those of the normal population due to the occurrence of pathological changes (Greicius et al., 2003).

At present, researchers have proposed many FCN models for disease diagnosis, which can be roughly divided into two categories. The first class is the so-called “low-order FCN” (Zhou et al., 2018b) that can only reflect FC characteristics between any two ROIs. For example, the conventional FCN assumes that all the rs-fMRI time series are static during the whole scanning period. Under such assumption, FC is quantified with the correlation (e.g., Pearson’s correlation) between a pair of rs-fMRI time series derived from two ROIs (Achard, 2006). The dynamic FCN overcomes the drawback that the conventional FCN cannot reflect the dynamic information of brain activity. Based on the sliding window strategy, the rs-fMRI time series are divided into a set of short time series fragments, and the conventional FCN is constructed on each fragment. This can capture dynamic FC changes over time to a certain extent (Kudela et al., 2017). Notice that the low-order FCNs only calculate the pairwise correlation between two brain ROIs while fail to reflect deeper linkage mechanism involving multiple ROIs inside the brain. And the functional connectivity involving multiple ROIs may contain complementary information to low-order FC. The second class of FCN model is the so-called “high-order FCN” (Song et al., 2020) that can capture deeper brain information by designing FC model of multiple ROIs. For example, on the basis of dynamic FCN, Chen et al. (2016) and Zhao et al. (2018) took each FC time series as the network node and the correlation coefficient of FC time series of each ROI pair as the edge weight to construct a high-order FCN, which fills the interaction between paired ROI and other ROI pairs. Zhang et al. (2016) proposed a novel method to capture second-level relationship between two brain regions using inter-regional resemblance of the FC topographical profiles, which complements the discovery of more biologically meaningful inter-group differences. Furthermore, Zhao et al. (2020) combined inter-regional resemblance of the FC topographical profiles with dynamic network and central moment to explore dynamic and high-order relationships between two brain regions, which mines the dynamic FC relationship of multiple ROIs from multiple perspectives. Of note, the above methods all share the “correlation’s correlation” strategy. In addition, in the literatures, many authors (e.g., Zhang et al., 2016, 2017) have presented the importance of high-order FC and explained potential biological meanings of high-order FC networks in dedicated studies. Since this paper mainly focuses on the applications of high-order FCNs for diagnosis, detailed discussion about general biological meanings of high-order FC networks can be found in these published works.

Zhou et al. (2018a) proposed a novel FC estimation method based on Matrix Variate Normal Distribution (MVND) theory. Compared with other higher-order models, MVND-based FCN can simultaneously obtain both low- and high-order FCNs with a clear mathematical explanation, and has demonstrated superior performance in identifying MCI patients from NCs. Specifically, the FCN sequence is constructed with the sliding window strategy, and then the so-constructed FCNs are taken as the samples to estimate the final low-order and high-order FCNs. In other words, each FCN is regarded as a random variable matrix (RVM) which obeys MVND, and all the FCNs in the sequence are taken together as the sample population to fit an MVND model. Like the other models mentioned above, Zhou’s work is an FCN construction method based on fully brain network (FBN). So, we use the term “fully network FCN method” to refer to the method presented by Zhou et al. (2018a).

However, the “fully network FCN method” has the problem of “high dimension but small sample,” which makes it actually impossible to fit an MVND model accurately. Theoretically, when fitting any distribution, the more samples there are, the more accurate the distribution will be. Besides, the higher spatial dimension where the distribution is located, the more samples will be needed in a fitting task. However, there exist the following facts in the “fully network FCN method”: (1) each FCN is represented as a 116 × 116 matrix. (2) Each rs-fMRI time series contains only 137 volumes at most leading that no more than 137 FCNs can be generated even through the sliding window strategy. In fact, it is almost impossible to fit such a high-dimensional distribution with such a small number of samples.

In general, for fitting a more accurate MVND, either reducing the dimension of the RVM or increasing the number of RVM will be helpful. In other words, the fitting accuracy of MVND can be improved by reducing the ratio between the dimension of RVM and the number of RVM. However, as mentioned above, it is impossible to generate more than 137 FCNs through sliding windows even in extreme cases, then increasing the number of RVM is not feasible. Therefore, we put forward the “hierarchical sub-network method” to improve the “fully network FCN method” from the perspective of reducing the dimension of RVM in this paper. Specifically, the brain network is divided into several sub-networks, and each sub-network contains only part of rs-fMRI time series. Firstly, the MVND based FCN construction algorithm is implemented in each sub-network, so as to reduce the spatial dimension of MVND and obtain more accurate estimates of intra-sub-network low- and high-order FCNs. Furthermore, the rs-fMRI mean series of all sub-networks are obtained, and the low- and high-order FCN across sub-networks are estimated according to the same strategy to compensate for the loss of FC information caused by sub-network division.

We propose the “hierarchical sub-network method” based on the following two motivations. On one hand, the ratio of the dimension of RVM to the number of RVM can be effectively decreased, so as to improve the fitting effect of MVND through the sub-network strategy. In fact, in this paper, the brain is divided into six relatively independent sub-networks according to the BrainNet Viewer software (Xia et al., 2013) like: the default mode network (DMN), the execution and attention network (EAN), etc. In other word, each sub-network is a relatively independent functional area and just contains a little part of ROIs. We take the largest sub-network as an example to illustrate the effectiveness of this method in improving MVND fitting. The largest sub-network only contains 26 ROIs, so in the MVND-based FCN construction method, RVM is expressed as a 26 × 26 matrix. Each ROI measured 170 signal elements, the ratio of the dimension of RVM to the number of RVM is 3.97 in our method, while in the “fully network FCN method,” as analyzed earlier, the RVM is represented as a 116 × 116 matrix and the dimension quantity ratio of RVM is 79.15. Therefore, our method can reduce the difficulty of MVND fitting from the perspective of spatial dimension.

**FIGURE 1:**
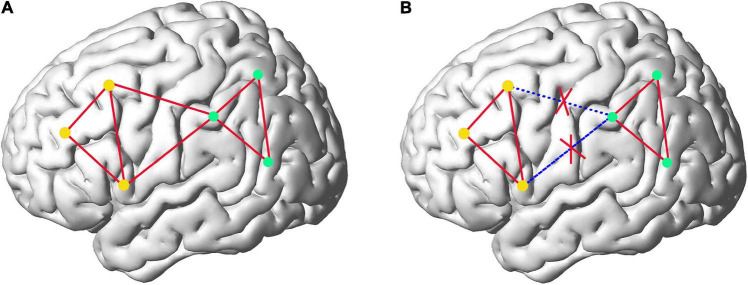
The comparison of the FC information captured in fully network **(A)** and sub-network **(B)** of brain. The dots represent ROIs and the red lines represent FCs. Yellow dots and green dots depict two different sub-networks.

On the other hand, although the sub-network strategy can achieve more accurate fitting of MVND and more accurate extraction of FC information in sub-networks, we have to point out the fact that merely building FCN in sub-networks inevitably loses FC information of ROIs across different sub-networks, which can be understood more clearly by comparing FC information captured in fully network (Figure 1A) and sub-network (Figure 1B) of brain. Figure 1A represents the eight FCs among the six ROIs before network division. Figure 1B reflects that the attention to FC information of ROI within the sub-network ignores the two FCs belonging to ROI of different networks. In this paper, we defuse this problem skillfully through the correlation of any pair of sub-networks. Specifically, we first average all the rs-fMRI time series in each sub-network to get 6 (the number of sub-networks) mean time series, and then take all sub-networks as nodes to construct the low- and high-order FCN with MVND based FCN construction method. Corresponding to intra-sub-network features, these features are called inter-sub-network features. Finally, both intra-sub-network features and inter-sub-network features are used as the basis for autism classification experiments.

In summary, the advantages of the “hierarchical sub-network method” are as follows: first, combining the MVND-based FCN construction method with functional sub-networks can reduce the spatial dimension of MVND and achieve more accurate fitting of MVND; Second, capturing intra-sub-network features and inter-sub-network features from macro and micro perspectives to achieve the full expression of FC information in brain networks. In order to verify that the “hierarchical sub-network method” is superior to the “fully network FCN method,” we apply both methods to the Autism Brain Imaging Data Exchange (ABIDE) database for individual based classification between ASD patients and NCs.

## Materials and Methods

### Data Acquisition and Preprocessing

In this study, 92 rs-fMRI images of subjects with ages ranging from 7 to 15 years old from the publicly available Autism Brain Image Data Exchange Database (ABIDE) (Di Martino et al., 2014) are used, including 45 ASD patients and 47 NCs In order to avoid the influences of the heterogeneity of multi-site data on the results due to the difference in medical device, collection protocol, etc., we chose 45 ASD patients (36 males and 9 females) and 47 NC subjects (36 males and 11 females) with ages ranging from 7 to 15 years old. The mean frame-wise displacement was computed to describe head motion for each individual. The individuals were excluded if their mean FD is larger than 1 mm (Lin et al., 2015; Ray et al., 2015). All these considered subjects had no excessive head motion with a displacement of < 1.5 mm or an angular rotation of < 1.5 in any of three directions. The detailed demographic information of these subjects is summarized in Table 1. As shown in Table 1, there are no significant differences (*p* > 0.05) in gender, age, and FIQ between two groups.

**TABLE 1:** Demographic information of the subjects.

Characteristic	NC	ASD	p-value
Gender (M/F)	36/9	36/11	0.2135^a^
Age (mean±*SD*)	11.1 ± 2.3	11.0 ± 2.3	0.773^b^
FIQ (mean ± *SD*)	106.8 ± 17.4	113.3 ± 14.1	0.0510^b^
ADI-R (mean ± SD)	32.2 ± 14.3^c^	-	-
ADOS (mean ± SD)	13.7 ± 5.0	-	-
FD (mm)(mean ± SD)	0.14 ± 0.05	0.15 ± 0.07	0.36^b^

*ASD, autism spectrum disorders; NC, normal control; M, male; F, female; FIQ, Full Intelligence Quotient; ADI-R, Autism Diagnostic Interview-Revised; ADOS, autism diagnostic observation schedule.*

*^a^Thep-value was obtained byχ^2^-test.*

*^b^The p-value was obtained by two-sample two-tailed t-test.*

*^c^Two patients do not have the ADI-R score.*

The observed rs-fMRI images are scanned at New York University (NYU) Langone Medical Center using a 3 -T Siemens Allegra scanner with the following parameters: flip angle = 90, 33 slices, TR/TE = 2,000/15 ms, 180 volumes, and voxel thickness = 4 mm. More details on the data collection, exclusion criteria, and scan parameters can be obtained from the ABIDE website.^1^

The acquired rs-fMRI data is preprocessed by the Statistical Parametric Mapping (SPM8) software.^2^ Then, the brain is parcellated into 116 ROIs using the Automated Anatomical Marker (AAL) atlas (Tzourio-Mazoyer et al., 2002), and the average rs-fMRI time series for each ROI are calculated and expressed as a data matrix *X* ∈ *R*^170×116^, where 170 denotes the total number of temporal image volumes and 116 denotes the total number of brain ROIs.

### The Pipeline of the “Hierarchical Sub-Network Method”

**FIGURE 2:**
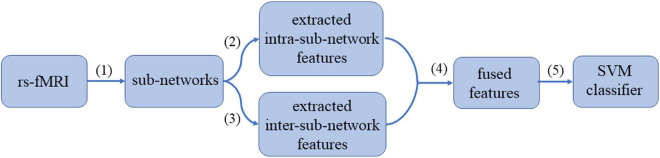
The pipeline of the “hierarchical sub-network method.”

The pipeline of our proposed “hierarchical sub-network method” is shown in Figure 2, which mainly includes the following four steps: (1) Sub-network division. The division labels of the sub-network are obtained according to the BrainNet Viewer software (Xia et al., 2013), and the rs-fMRI time series of each subject are divided into 6 groups according to the division labels. (2) Intra-sub-network feature extraction. In each sub-network, the FCN sequence is constructed with sliding window, and the MVND is fitted with the FCN sequence being the RVM sample to obtain the intra-sub-network features. (3) Inter-sub-network feature extraction. The mean time series of each subnetwork is calculated, and then the low-order and high-order FCNs of the fully network are estimated synchronously with the MVND-based FCN construction method. (4) Feature normalization, feature selection and feature fusion. The features obtained in steps (2)—(3) are normalized. Then we use *T*-test and LASSO algorithms to select the most relevant features for the classification task. (5) ASD classification. We use SVM with linear kernel for ASD classification.

In the following subsections, we describe the above steps in detail. The meanings of the mathematical symbols are that bold uppercase letters represent matrices (i.e., **M**), regular uppercase letters represent total values (i.e.,*M*), bold lowercase letters represent vectors (i.e., **m**), and regular lowercase letters represent scalars (i.e., *m*).

### Dividing the Brain Into Sub-Networks

**FIGURE 3:**
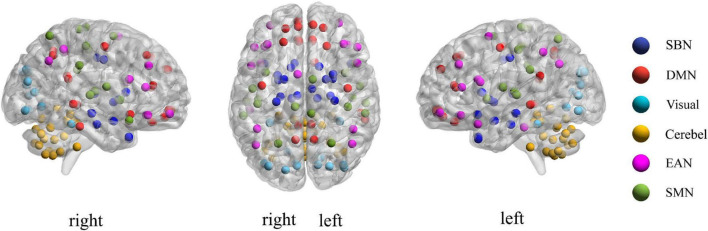
Visualization of the location of each sub-network in brain.

For each subject, we define **x**_*i*_ = (*x*_*i*1_, *x*_*i*2_, ⋯, *x*_*iM*_)(*i* = 1,2, ⋯,*N*) as the average rs-fMRI time series across all voxels belonging to the *i*-th ROI, where *M* denotes the total number of temporal image volumes, and *N* denotes the total number of ROIs. According to the experimental data mentioned above, here *M* = 170 and *N* = 116. Divide all ROIs into *U* different sub-networks {Ω_1_,Ω_2_,⋯,Ω_*u*_,⋯,Ω_*U*_}, where *U* consists of index *i* if *x_i_* is included in the *u*-th sub-network. In the current study, the 116 ROIs in the Automated Anatomical Labeling (AAL) template were divided into six common functional networks according to the BrainNet Viewer software (Xia et al., 2013): the default mode network (DMN), the execution and attention network (EAN), the sensorimotor network (SMN), the visual network (Visual), the subcortical nuclei (SBN) regions and the cerebellum (Cerebel), so here *U* = 6. Of note, we choose this division method for the following two reasons. On one hand, the generated six sub-networks based on the BrainNet Viewer software have clear biological explanation, which makes this study have a broader medical reference value. On the other hand, dividing six sub-networks is enough to satisfy the dimensionality reduction needs of this study. Since the number of ROIs varies in each sub-network, we can use *N_u_* to denote the total number of ROIs in the *u*-th sub-network. Figure 3 gives an intuitive view of the division of the sub-network. In section “Discussion,” we also discuss the sub-network division method based on similarity.

### Constructing the Functional Connectivity Network Time Series With Sliding-Window Strategy

**FIGURE 4:**
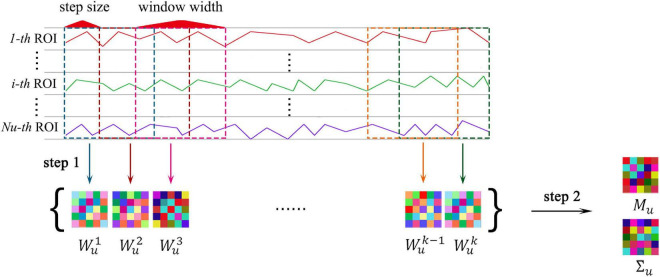
The MVND based FCN construction method. Where, **step 1** shows the sliding-window strategy, **step 2** shows the MVND based feature extraction.


In Figure 4, **step 1** illustrate the construction of the FCN time series with sliding-window strategy vividly. Let the correlation between the *i*-th and the *j*-th ROIs be:

(1) $$c_{i\,j}=c\,o\,r\,r\,\left({\text{x}}_{i},{\text{x}}_{j}\right)$$

Then, an FCN can be established using the classical method by taking **x_i_** as nodes and *c*_*ij*_ as weights of edges. Here, *c*_*ij*_ is the weight of the edge connecting the *i*-th ROI and the *j*-th ROI. In the *u*-th sub-network, *i*,*j* ∈ Ω_*u*_, the total number of nodes of FCN is *N_u_*, thus FCN can be expressed as a symmetric matrix, defined as follows:


(2) $${\text{W}}_{u}=\left(c_{i\,j}\right)\,i,j\in \Omega_{u}$$

where **W**_*u*_ ∈ *R^N_u_^* ^×^^*N_u_*^ represents the FCN in the *u*-th sub-network. Next, the sliding window strategy is introduced. The entire rs-fMRI time series of all ROIs is divided into *K* segments by window sliding, and corresponding FCNs are established on each rs-fMRI time series segment, thus forming a sequence containing *K* FCNs where *K* is determined by the window width *l_w_* and step size *l_s_* of the sliding window.

Specifically, taking the *u*-th sub-network as an example, the total number of ROIs is *N_u_*, the total number of timing image voxels is *M*, and a sequence containing *K* FCNs, denoted by $\left\{{\text{W}}_{u}^{1},{\text{W}}_{u}^{2},\cdots ,{\text{W}}_{u}^{K}\right\}$, will be obtained through the sliding window strategy, where $K=\left[\frac{M-l_{w}}{l_{s}}\right]+1,M=170$, *l*_*w*_ and *l_s_* are variable parameters.

### Extracting the Intra-Sub-Network Features

**In** Figure 4, step 2 displays the pipeline of the extraction of intra-sub-network features. In each sub-network, we regard the obtained FCN sequence as a sample population obeying a multivariate Gaussian distribution, then each FCN is regarded as a random variable matrix sample, then


(3) $${\text{W}}_{u}^{k}∼N\,\left({\text{M}}_{u},\Sigma_{u}\right)\,1\leq k\leq K$$

where, **M**_*u*_ ∈ *R^N_u_^* ^×^^*N_u_*^ is the population mean or mathematical expectation, and $\Sigma_{u}\in R^{N_{u}^{2}\times N_{u}^{2}}$ is the population covariance of **W**_*u*_. As mentioned in the introduction, **M**_*u*_ and **Σ***_u_* correspond to the low-order FC Features and high-order FC Features of brain networks, respectively. Since the dimension of **Σ***_u_* is too high, in order to avoid overfitting in the classification experiment, and consistent with the method in Zhou et al. (2018a) we replace the population variance with the form of Kronecker product decomposition (Gupta and Nagar, 2000), i.e., $\Sigma ={\text{C}}_{u}^{1}⊗{\text{C}}_{u}^{2}$, where ${\text{C}}_{u}^{1},{\text{C}}_{u}^{2}\in R^{N_{u}\times N_{u}}$ are positive semi-definite, representing the column and row covariance matrices of **W**_*u*_, respectively. Since **W**_*u*_ is a symmetric matrix, ${\text{C}}_{u}^{1}={\text{C}}_{u}^{2}$, we can use ${\text{C}}_{u}={\text{C}}_{u}^{1}={\text{C}}_{u}^{2}$ to replace **Σ***_u_* with the advantage of not losing information, so as to achieve the dimension reduction of **Σ***_u_*. Specifically, according to maximum likelihood estimation (MLE) theory of MVND, in each sub-network, the MLE of **M**_*u*_ is


(4) $${\text{M}}_{u}=\frac{1}{K}\,\sum_{k=1}^{K}{\text{W}}_{u}^{k}$$

The MLE of **C**_*u*_ can be achieved by the following iteration formula:


(5) $${\text{C}}_{u}=\frac{1}{K\,N_{u}}\,\sum_{k=1}^{K}\left({\text{W}}_{u}^{k}-{\text{M}}_{u}\right)\,{\text{C}}_{u}^{-1}\,{\left({\text{W}}_{u}^{k}-{\text{M}}_{u}\right)}^{T}$$

where, 1 ≤ *k* ≤ *K*, 1 ≤ *u* ≤ *U*.

### Extracting the Inter-Sub-Network Features

**FIGURE 5:**
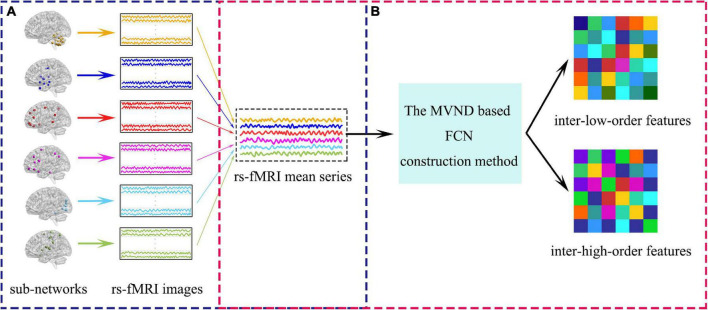
The overview of the extraction of inter-sub-network features. **(A)** Shows the calculation of rs-fMRI mean series. **(B)** Shows the pipeline of the extraction of the inter-sub-network features.

As mentioned in the introduction, after the sub-network division, we must consider both intra-sub-network and inter-sub-network features. The overview of the extraction of inter-sub-network features is vividly illustrated in Figure 5 and the extraction of inter-sub-network features is divided into two steps: (1) Calculating the mean correlation time series for each sub-network (see Figure 5A). (2) Estimating low- and high-order FCNs simultaneously with the MVND based FCN construction method from rs-fMRI mean time series (see Figure 5B). The estimated low- and high-order FCNs are the inter-low-order features and the inter-high-order features, respectively.

The inter-sub-network feature extraction method is equivalent to the construction of FCN in the whole brain scale and the FCN construction method is the same as that in the sub-network scale. Both are constructed by MVND based FCN construction method, which can be referred to section “Constructing the FCN Time Series With Sliding-Window Strategy” and section “Extracting the Intra-Sub-Network Features.” Here we describe in detail the generation of mean time series of each sub-network by taking the *u*-th subnetwork as an example.

The mean correlation time series **y_u_** of the *u*-th sub-network can be calculated by averaging those rs-fMRI time series assigned to this sub-network. Specifically, each element in **y**_*u*_ is defined as:


(6) $${\text{y}}_{u}^{m}=\frac{\sum i\in \Omega_{u}\,{\text{y}}_{i}^{m}}{||\Omega_{u}||},1\leq m\leq M$$

Where, *m* represents the subscript of the element in **y**_*u*_, and |||u| represents the total number of rs-fMRI time series contained in the *u*-th sub-network.

### Feature Normalization, Selection, Fusion, and Classification

All the features we have obtained include the intra-sub-network features and the inter-sub-network features, each of which consists of both high-order features and low-order features. Let’s call them **f_1_**,**f_2_**,**f**_3_,*and***f**_4_. These four feature vectors are acquired in different ways, so there are inevitably scale differences among different features. In order to treat each feature equally, we normalize them in the same way. Here, **f**_1_,**f**_2_,**f**_3_,*and***f**_4_ are normalized by the “min-max normalization” method, respectively. Take **f**_1_ as an example:


(7) $${\text{f}}_{1\,i}^{\prime }=\frac{{\text{f}}_{1\,i}-\text{min}\,\left({\text{f}}_{1}\right)}{\max\left({\text{f}}_{1}\right)-\text{min}\,\left({\text{f}}_{1}\right)}$$

Where, **f**_1_ represents the vector of intra-sub-network high-order features. **f**_1*i*_ represents the *i*-th element in **f**_1_, *min*(**f**_1_) represents the minimum value in **f**_1_, and *max*(**f**_1_) represents the maximum value in **f**_1_. The four types of features obtained by the “sub-network FCN method” just reflect the functional connectivity relationship between or among ROIs from four perspectives and they are complementary and homogeneous. Therefore, our fusion method is to simply combine them as a whole. In other words, the normalized feature data of four feature vectors are concatenated and expressed as a long vector f, that is $\text{f}=\text{[}f_{1}^{\prime },f_{2}^{\prime },f_{3}^{\prime },f_{4}^{\prime }]$.

However, the intra-sub-network low-order features and the inter-sub-network low-order features expressed by **f**_2_ and **f**_4_ exists as the form of FCN. FCN is a symmetric matrix, and the repeated feature leads to redundancy. So, we vectorize their lower off-diagonal-triangular parts to redefine the feature vectors. In this way, the original feature represented by **f** is replaced by a new one denoted by **f**_*a*_. Obviously, **f**_*a*_ may still contain features unrelated to ASD disease. In order to reduce the interference of irrelevant features and improve the generalization performance, we use the two-stage feature selection strategy to select a small set of most discriminative features for ASD diagnosis.

The first step is to perform a two-sample *t*-test between NCs and ASD subjects for each feature in the **f**_*a*_. Those features whose *p*-value is smaller than a certain threshold are preserved. At this point, we label the newly obtained feature set as **f**_*b*_. In the second step, we apply the *L_1_*-norm regularized least squares regression, known as *LASSO* (Tibshirani, 1996), to further select the discriminative features from **f**_*b*_. Specifically, we used ${\text{f}}_{b}^{l}$ to denote the features of the*l*-th subject and *I^l^* to represent the label of the *l*-th subject. If the *l*-th subject is a patient with ASD, *I^l^* = 1; otherwise, *I^l^* = −1. Let **w** represents the weight vector for the feature selection task. The *LASSO* model is expressed by mathematical formula as:


(8) $$\min\frac{1}{2}\,\sum_{l=1}^{L}{||I^{l}-{\left({\text{f}}_{b}^{l}\right)}^{T}\,\text{w}||}_{2}^{2}\,\lambda \,{||w||}_{1}$$

Where, *L* represents the total number of subjects, and*L* = 92 in this experiment. λ is a parameter, controlling the model’s sparsity based on the *L_1_*-norm regularization. The larger the value of λ, the sparser the model is. Different from the *t*-test, which selects feature separately,*LASSO* investigates all features synchronously. The *t*-test method and the LASSO method select features from different perspectives. As a binary classification problem, the *t*-test method can effectively select the features with high significance in ASD subjects and NC subjects. However, *t*-test method treats each feature independently without taking into account their inherent correlation, thus possibly resulting in many redundant features. Therefore, we further use LASSO method for the second selection which is able to consider the relationship between features. Therefore, we combine the two methods and design a two-stage feature selection strategy. We use **F** to represent the final feature for classification. In the classification phase, we use SVM (Chang and Lin, 2011) with a simple linear kernel for ASD identification. SVM seeks a maximum margin hyper-plane to separate the two kinds of samples. By adjusting the hyperparameter γ, the empirical risk of the training data and the complexity of the model can be balanced, so as to obtain good generalization performance on unlabeled test data.

### Evaluation Methodology

We use nested fivefold cross-validation strategy which consists of two nested loops to evaluate classification performance in this experiment. In outer loop, 92 subjects are divided into 5 subsets of the roughly same size, where one subset is selected as the test-set, and the other 4 subsets are used as the training-set. In inner loop, the data of the training-set are combined and redivided into five subsets of similar size, four of which are used for tuning the hyperparameters and one for model evaluation. The performance of our method is mainly affected by three hyperparameters, they are p and λ in feature selection and γ in SVM model. The optimal hyperparameters can be determined when the average classification accuracy reaches its optimum. we determine the optimal values for the parameters in the following range: p ∈ [0.01:0.01:0.1],λ ∈ [0.1:0.1:0.9], and γ ∈ [2^−4^,⋯,2^4^]. When the optimal hyperparameters are selected in inner loop, they are returned to the outer loop where the model will be trained based on the training dataset and evaluated on the testing dataset. Besides classification accuracy (ACC), we use sensitivity or true positive rate (TPR), specificity or true negative rate (TNR), positive predictive value (PPV), and negative predictive value (NPV)^3^ to comprehensively evaluate the classification performance of the two methods.

## Results

### Autism Spectrum Disorder Classification Performance

In this work, we compare the performance of the “hierarchical sub-network method” and the “fully network FCN method” in the ASD classification experiment. Specifically, we use the fusion of all the features extracted by each method to perform classification experiments. The experimental results are shown in Table 2 and can be found with *Sub-Fusion* and *Fully Fusion* as pointers. Furthermore, in order to analyze the influence of different types of features in two compared methods on the experimental results, we carry out separate experiments on intra-sub-network high-order features, intra-sub-network low-order features, inter-sub-network high-order features, inter-sub-network low-order features, fully network high-order features and fully network low-order features. In Table 2, they are abbreviated as *Sub-Intra-High*, *Sub-Intra-Low*, *Sub-Inter-High*, *Sub-Inter-Low*, *Fully High*, and *Fully Low*. In addition, we conduct experiments on the fusion of intra-sub-network features and inter-sub-network features in the “hierarchical sub-network method” and the results can be found with the pointer *Sub-Intra-Fusion* and *Sub-Inter-Fusion* in Table 2. Finally, we experimented with two traditional methods under the same data, and reported the experimental results in Table 2. Traditional static FCN method and low-order dynamic FCN method are abbreviated as *con-static* and *con-dynamic*, respectively, in Table 2.

**TABLE 2:** ASD classification performance using different features.

Feature type	ACC (%)	TPR (%)	TNR (%)	PPV (%)	NPV (%)
*Sub-Intra-Low*	74 ± 0.21	73 ± 0.33	75 ± 0.39	75 ± 0.38	73 ± 0.49
*Sub-Intra-High*	77 ± 0.30	73 ± 0.45	81 ± 0.14	79 ± 0.47	76 ± 0.12
*Sub-Intra-Fusion*	79 ± 0.49	76 ± 0.45	82 ± 0.23	81 ± 0.13	77 ± 0.25
*Sub-Inter-Low*	66 ± 0.30	62 ± 0.22	70 ± 0.21	67 ± 0.45	66 ± 0.00
*Sub-Inter-High*	72 ± 0.38	69 ± 0.22	74 ± 0.40	72 ± 0.09	71 ± 0.43
*Sub-Inter-Fusion*	73 ± 0.45	71 ± 0.45	72 ± 0.34	71 ± 0.11	72 ± 0.34
*Sub-Fusion*	**81** ± **0.44**	**78** ± **0.30**	**83** ± **0.11**	**81** ± **0.45**	**80** ± **0.44**
*Fully Low*	74 ± 0.30	78 ± 0.29	70 ± 0.30	71 ± 0.45	77 ± 0.37
*Fully High*	71 ± 0.45	65 ± 0.37	77 ± 0.45	72 ± 0.50	69 ± 0.25
*Fully Fusion*	75 ± 0.18	72 ± 0.34	74 ± 0.48	74 ± 0.22	73 ± 0.14
*Con-static*	74 ± 0.04	72 ± 0.23	76 ± 0.01	74 ± 0.05	73 ± 0.07
*Con-dynamic*	75 ± 0.12	73 ± 0.14	76 ± 0.29	74 ± 0.23	75 ± 0.08

*Values highlighted in bold mean the best results.*

Table 2 shows the mean classification performance for each compared feature type. From the experimental results shown in Table 2, we can make the following judgments: (1) The classification accuracy of the intra-sub-network low- and high-order features of the “hierarchical sub-network method” (i.e., *Sub-Intra-Low*, *Sub-Intra-High*) is better than the corresponding features extracted by the “fully-network FCN method” (i.e., *Fully-Low*, *Fully-High*). (2) Both in the “hierarchical sub-network method” and the “fully-network FCN method”, the classification performance of fusion features is significantly better than those of each type of features alone. (3) The performance of the fusion features (i.e., *Sub-Fusion*) of the “hierarchical sub-network method” is significantly higher than those (i.e., *Fully-Fusion*) of the “fully-network FCN method”. (4) The classification performance of the fusion of the Intra-sub-network features extracted by “hierarchical sub-network method” (i.e., *Sub-Intra-Fusion*) is significantly better than the fusion of features extracted by “fully-network FCN method”(i.e., *Fully-Fusion*). (5) Both the “fully-network FCN method” and the “hierarchical sub-network FCN method” perform better than the two traditional FCN methods, and the “hierarchical sub-network FCN method” has the most obvious advantages.

### Influence of Parameters on Accuracy

**FIGURE 6:**
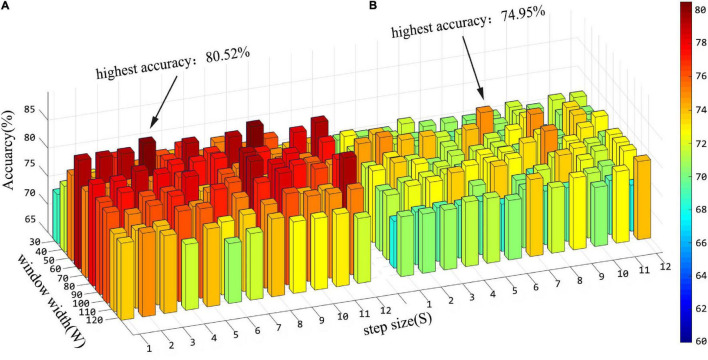
Histogram of classification accuracy of the “hierarchical sub-network method” **(A)** and the “fully-network FCN method” **(B)** under various sliding window parameters.

In the “hierarchical sub-network method”, we use the sliding window strategy to generate FCN sequences. There are two key parameters of the sliding window strategy that have a crucial impact on feature extraction and further affect the final recognition accuracy. They are the window width **(W)** and the step size **(S)** of the sliding window. In order to evaluate the influence of these two parameters on the experimental results, we conducted an ASD classification experiment under different parameter combinations. The window width is set as [30:10:120] and the step size is set as [1:1:12]. Figure 6 shows the average accuracy of ASD classification under different parameter combinations. Referring to Figure 6, we can draw the following conclusions: (1) Sliding window parameters have great influence on classification performance. In the “hierarchical sub-network method,” the maximum recognition accuracy is obtained when the window width is 60 and the step size is 4; The best performance of the “fully network FCN method” is achieved when the window width is 50 and the step size is 7. (2) In the performance comparison between the “hierarchical sub-network method” and the “fully network FCN method” under the same sliding window parameters, the “hierarchical sub-network method” is superior to the “fully network FCN method” in the majority of cases. (3) In each method, the best classification performance is achieved on average when the window width is between 50 and 90.

**FIGURE 7:**
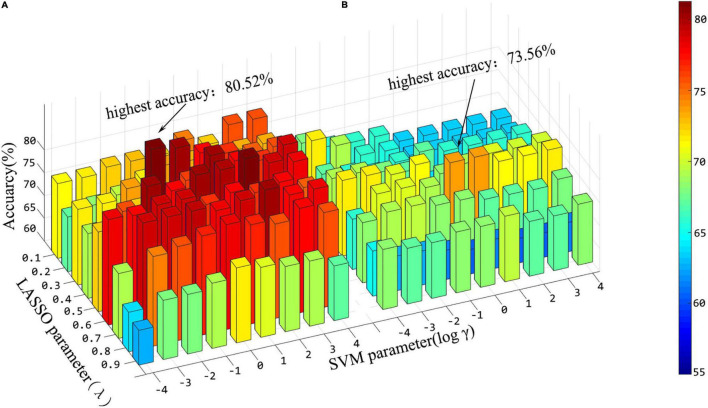
Histogram of classification accuracy of the “hierarchical sub-network method” **(A)** and the “full-network FCN method” **(B)** under various hyper-parameter combinations.

In addition, in the stage of feature fusion and classifier training, three hyperparameters have great impact on the results, that is, *p*-values in *t*-test, λ in lasso and γ in SVM. In this experiment, we explored the effects of different combinations of λ and γ on the results. Before that, the window width and step size are fixed as 60 and 4, respectively, which are also the parameter when the “hierarchical sub-network method” reaches the maximum. Figure 7 shows the classification accuracy under different combinations of λ and γ in the two methods when the hyperparameter *p* = 1 of the *t*-test. From Figure 7, we can see that hyperparameter λ and γ have significant influence on the experimental results, and the effects are different in the two experiments.

### The Most Discriminative Sub-Networks and Features for Autism Spectrum Disorder Diagnosis

**FIGURE 8:**
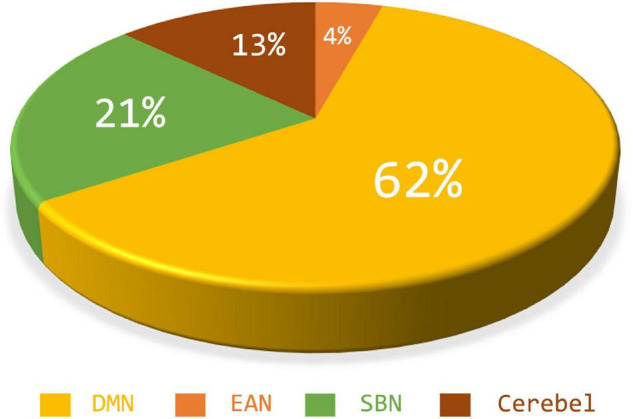
The feature contribution rate of different sub-networks to classifier training.

**FIGURE 9:**
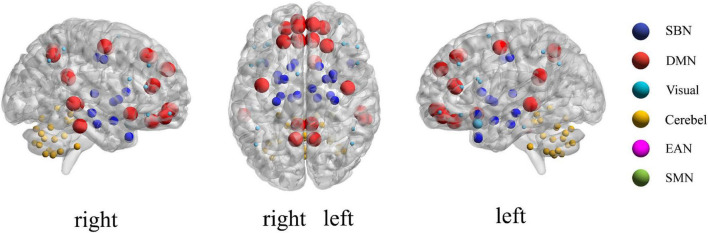
The sub-network associated with ASD and the strength of its contribution to ASD.

According to the feature selection method mentioned in ***Feature normalization, selection, and classification*,***t*-test and*LASSO* are used to extract the most discriminative features from the original features in two steps for the ASD classification experiment. From all fivefold validation experiments, we take out and analyze the features used for training classifier each time. We trace each feature to each sub-network and count how often each sub-network is tracked. According to the frequency, the contribution of each sub-network to ASD recognition is calculated. The higher the frequency, the greater the contribution of the sub-network. Figure 8 shows the contribution and distribution of different sub-networks. In order to have a more vivid and deep impression, the distribution of sub-network contribution is displayed on a surface rendering of the brain using the BrainNet viewer software (see Figure 9). The larger the volume of the ball, the greater the contribution rate of the sub-network to ASD recognition. Each sphere represents an ROI, we only use the set of spheres with the same color to represent the sub-network to observe the relationship between each sub-network and ASD.

**FIGURE 10:**
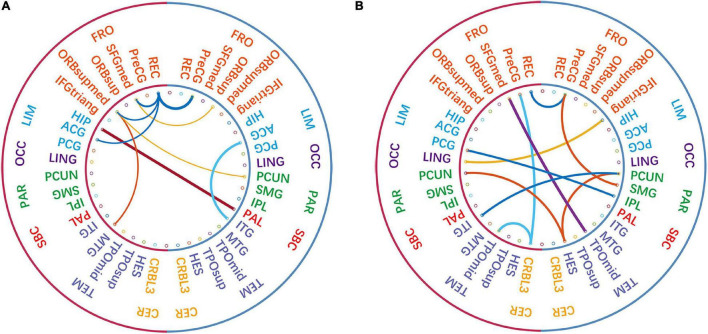
The comparison of the most discriminative features in the “hierarchical sub-network method” **(A)** and the “fully-network FCN method” **(B)**. The left-side of each diagram represents the left hemisphere of the brain, and the right-side represents the right hemisphere of the brain. In the inner circle, each line connects two ROIs, and the thickness of the line represents the strength of its identification ability.

Combined with Figures 8, 9, we can see that only four sub-networks provide discriminative features for classification experiments, and DMN is the sub-network that contributes the most. This suggests that sub-network DMN is closely related to the diagnosis of ASD. Sub-network SMN and Visual provide zero contribution in this study, and precise judgments need further research. In addition, we believe that tracing the FC features that contribute most is also a convincing perspective to compare the differences between the two methods. The intra-sub-network low-order FCN and the fully network low-order FCN are used in the classification experiment. Then *t*-test and*LASSO* regression are used to select the features twice to get the final features for training. This part of the feature is considered the most discriminating. Each of these features represents an FC between a pair of ROIs. The features extracted in 10 repeated experiments are counted, and the top 10 features with the highest frequency are selected and shown in Figure 10. The name of the ROIs and brain anatomic areas shown in Figure 10 are referred to the file (“Node\_AAL116.node”) provided by BrainNet Viewer software.

Although certain genes have been found to be involved in ASD, the affected brain regions and the mechanisms behind specific defects are still poorly understood. According to Figure 10, except that the functional connections of REC in the left FRO region and PreCG in the right FRO region are selected by both methods, other features are different. The functional connection features selected by the “hierarchical sub-network method” are mostly concentrated in the FRO region. In fact, current studies have confirmed the relationship between FRO lesions and ASD disease (Scott-Van Zeeland et al., 2010; Solso et al., 2016).

## Discussion

We proposed “hierarchical sub-network method” based on MVND theory. This method not only inherits the advantages of MVND based FCN construction, being able to simultaneously obtain high-order features reflecting FC information among multiple ROIs and low-order features reflecting FC information between any two ROIs, but also improves the fitting effect of MVND with the help of sub-network division, so as to capture the functional connections of the brain more accurately and provide more discriminative features. We believe that compared with the “fully network FCN method,” the “hierarchical sub-network method” can fully mine the disease-disturbed FCN variation information and has a better performance in ASD classification experiments. We will give a more detailed discussion on the comparison of the two methods.

In order to have an intuitive understanding of the dimension of features extracted by the “hierarchical sub-network method” and the “fully network FCN method,” we select the dimension of intra-sub-network low-order features and fully network low-order features as the representative to display. In detail, we set the window width parameter as 60 and the step size parameter as 1, which is the combination of sliding window parameters when the intra-sub-network-low-order features have the best performance in ASD classification experiment.

**FIGURE 11:**
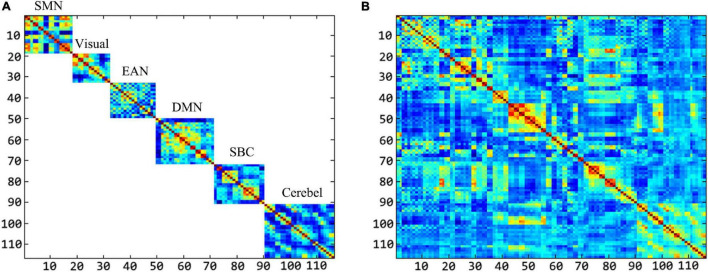
The comparison of the feature dimension in both methods. **(A)** Shows the feature dimension of each sub-network. **(B)** Shows the feature dimension of the fully-network.

We can take the area of the feature image as a reference to perceive the dimension of the feature extracted by the two methods. The larger the area, the higher the dimension. In this experiment, the number of ROI in each sub-network is as follows: 18 in SMN, 14 in Visual, 17 in EAN, 22 in DMN, 19 in SBC, and 26 in Cerebel. Each sub-network is shown in order from top to bottom in Figure 11. We can see that the intra-sub-network low-order feature dimensions extracted by different sub-networks are, respectively, about 2.41, 1.45, 2.15, 3.60, 2.68, 5.02% of the fully network low-order feature dimensions. Therefore, the “high dimensional but small-sample of RVM” problem is greatly improved in the calculation of fitting multivariate Gaussian distribution in each sub-network. Since the MVND based FCN construction method generates a high-order FCN and a low-order FCN with the same dimensions at one time, the dimension comparison of the high-order FCNs in the two methods is similar to that of the low-order FCNs. Overall, intra-sub-network features are more concise and discriminative than the fully network features.

The results of ASD classification experiments show that: (1) when the high-order and low-order features extracted by the “hierarchical sub-network method” are trained separately, the classification accuracy is higher than that of the “fully network FCN method,” and the classification performance of the “hierarchical sub-network method” is better than that of the “fully network FCN method.” This means that the “hierarchical sub-network method” can capture the FC changes more accurately. There are two factors that play a role together: first, in technology, the dimensionality of the FCN is reduced due to the division of the sub-network, i.e., the dimension decline of RVM, which makes the MVND fitting more accurately. Another factor is about the biological mechanism. The sub-network division of the brain integrates the ROIs which are closely related in function, and focuses on observing the functional connection relationship among the ROIs belonging to the same sub-network, so that the information reflected by the RVM is more concise and effective, and the extracted low-order features and high-order features have a more discriminative power in ASD classification experiment. (2) The fusion of features in each method leads to better classification performance, respectively. To be sure, high-order features and low-order features can provide complementary information, which is the reason why the fusion features have better classification performance in ASD classification experiments.

In the experiment to explore the influence of sliding window parameters on the classification accuracy, we found that the classification results of the two methods were changed with the combination of window width and step size parameters, especially the window width. Short window width and long window width have their own advantages and disadvantages. Short window width can provide rich short-term dynamic change information, but it is not stable due to the lack of low-frequency cycle (Sakoğlu et al., 2010). Long window width can make FC estimation more robust (Wang et al., 2018). From Figure 6, we can see the phenomenon that the maximum classification accuracy can be achieved in the middle length of the window width. In addition, under the same parameter combination, the “hierarchical sub-network method” is almost always more accurate than that of the “fully network FCN method.” Since the dimension of the RVM is lower in each sub-network, more accurate fitting can be obtained. This proves in practice that the superiority of the “hierarchical sub-network method” is not a special result under special conditions.

To further demonstrate the validity of the “hierarchical sub-network method” in the diagnosis of ASD disease, we trace the sub-network to which the features of the classifier used for training belong. The experimental results showed that features in DMN was selected most frequently, suggesting that DMN was closely related to the pathogenesis of ASD. We found that this conclusion was basically consistent with the conclusion of other studies (Rutter et al., 2009; Simon and Engstrom, 2015; Churchill et al., 2018), and abnormalities of the DMN were commonly regarded as prominent ASD neurobiological features (Padmanabhan et al., 2017). From a biomedical perspective, DMN plays a crucial role in socially related stimuli because it is involved in the mental state of self-reflective thinking and considering the perspective of others, which is consistent with the fact that ASD is characterized by difficulties in social communication and interaction (Padmanabhan et al., 2017). Some studies have reported that the widely decreased of the FC in DMN in ASD not only contributes to the core defect of ASD, but also has a significant impact on the symptom severity (Assaf et al., 2010; Weng et al., 2010; Kiselev, 2014). For example, Assaf et al. (2010) pointed out that the decrease of functional connectivity in DMN of ASD patients was negatively correlated with the severity of social and communication disorders.

In this study, we choose the medical template as the framework of sub-network division because of its advantage of biological interpretation. Of note, this is not the only scheme for sub-network partition. For example, ROI grouping based on the similarity of rs-fMRI time series can also be used as a sub-network division method. Specifically, k-means algorithm is used to cluster rs-fMRI time series, and the number of sub-networks is determined by specifying the number of clusters. By fixing the number of clusters from 6 to 11, we try to apply the similarity based sub-network division as an alternative to the “hierarchical sub network,” and verify it in the classification experiments of ASD and NC. When the number of clusters is fixed at 8, the classification accuracy gets maximum, and the experimental results are shown in Table 3. In order to distinguish from the existing features, we use “*intra-cluster*” and “*inter-cluster*” to represent the intra-sub-network and inter-sub-network features in this method, respectively, and “*Cluster-Fusion*” to represent the fusion features. From the results, the random division of sub-networks according to the similarity of rs-fMRI time series does not perform better than the existing methods, and as far as we know, this method has two obvious shortcomings: first, although it shows better performance, it cannot make a biological explanation for the results. Second, the number of clusters is not easy to determine which is greatly affected by subjects.

**TABLE 3:** Experimental results of the application of similarity based sub-network division.

Feature type	ACC (%)	TPR (%)	TNR (%)	PPV (%)	NPV (%)
*Intra-cluster*	73 ± 0.91	77 ± 0.78	70 ± 0.21	71 ± 0.43	76 ± 0.74
*Inter-cluster*	61 ± 0.96	55 ± 0.56	68 ± 0.09	62 ± 0.50	61 ± 0.54
*Cluster-Fusion*	78 ± 0.26	77 ± 0.78	78 ± 0.72	77 ± 0.78	75 ± 0.72

## Conclusion

This paper proposes a new strategy for mental illness diagnosis based on FCN. The proposed method is based on the following two considerations: Technically, the FCN based on MVND is not well constructed in the fully network domain, and there exists the problem of “high dimension but small sample of RVM.” From the biological point of view, many mental diseases reflect the sub-network property of brain function, and the aggregation of functional linkage makes the diagnosis of diseases more targeted. The results of ASD classification experiments show that the “hierarchical sub-network method” is comparable to the “fully network FCN method,” and the biomedical findings obtained are consistent with other studies.

Besides Pearson’s correlation, we can also utilize other candidates, such as Flexible Least Squares (FLS) method provided by the DynamicBC toolbox, to construct low-order FC network. In comparison with Pearson’s correlation, FLS method has the advantage that more dynamic FC networks can be calculated by avoiding the sliding-window approach. The influence of different low-order FC networks to the performance of high-order FC network will be one of our directions for further study. One limitation of this work is that the ROIs corresponding to the higher-order features cannot be traced in the ASD classification experiment and this makes higher-order features useless for the discovery of ASD lesions. Further exploration of physiological markers of ASD and effective algorithms is our future work. Another limitation is that the “hierarchical sub-network method” could only explore the network-wise inter-network FCs, but would miss the ROI-wise inter-network FCs. How to compensate for the lost FCs with ROI-wise inter-network FCs needs to be further explored.

## Data Availability Statement

The original contributions presented in the study are included in the article/supplementary material, further inquiries can be directed to the corresponding author/s.

## Ethics Statement

The study was reviewed and approved by the Ethics Committee of Shandong Technology and Business University. All procedures performed in studies involving human participants were in accordance with the ethical standards of the institutional and/or national research committee and with the 1964 Helsinki declaration and its later amendments or comparable ethical standards.

## Author Contributions

FZ: conceptualization, methodology, and writing—review and editing. ZH: conceptualization, software, writing—original draft, methodology, formal analysis, investigation, and validation. DC, NM, and YL: writing—review and editing. XC and DF: conceptualization and writing—review and editing. PL: writing—review and editing, investigation, and supervision. All authors contributed to the article and approved the submitted version.

## Conflict of Interest

The authors declare that the research was conducted in the absence of any commercial or financial relationships that could be construed as a potential conflict of interest.

## Publisher’s Note

All claims expressed in this article are solely those of the authors and do not necessarily represent those of their affiliated organizations, or those of the publisher, the editors and the reviewers. Any product that may be evaluated in this article, or claim that may be made by its manufacturer, is not guaranteed or endorsed by the publisher.

## Footnotes

1
http://fcon_1000.projects.nitrc.org/indi/abide/abide_I.html

2
http://www.fil.ion.ucl.ac.uk/spm/software/spm8/

3
https://en.wikipedia.org/wiki/Sensitivity\_and\_specificity
